# Mechanistic Insight of Allantoin in Protecting Tomato Plants Against Ultraviolet C Stress

**DOI:** 10.3390/plants10010011

**Published:** 2020-12-23

**Authors:** Mona F. A. Dawood, Md. Tahjib-Ul-Arif, Abdullah Al Mamun Sohag, Arafat Abdel Hamed Abdel Latef, Marwa M. Ragaey

**Affiliations:** 1 Botany and Microbiology Department, Faculty of Science, Assiut University, Assiut 71516, Egypt; mo_fa87@aun.edu.eg; 2 Department of Biochemistry and Molecular Biology, Faculty of Agriculture, Bangladesh Agricultural University, Mymensingh 2202, Bangladesh; tahjib@bau.edu.bd (M.T.-U.-A.); sohag2010bmb.sust@gmail.com (A.A.M.S.); 3 Department of Biology, Turabah University College, Turabah Branch, Taif University, P.O. Box 11099, Taif 21944, Saudi Arabia; 4 Botany and Microbiology Department, Faculty of Science, South Valley University, Qena 83523, Egypt; 5 Botany and Microbiology Department, Faculty of Science, New Valley University, Al-Kharja 72511, Egypt; marwa_ragaey@sci.nvu.edu.eg

**Keywords:** allantoin, antioxidant enzymes, oxidative stress, secondary metabolites, tomato, ultraviolet stress

## Abstract

Allantoin ((AT) a purine metabolite)-mediated ultraviolet C (UVC) stress mitigation has not been studied to date. Here, we reported the physicochemical mechanisms of UVC-induced stress in tomato (*Solanum lycopersicum* L.) plants, including an AT-directed mitigation strategy. UVC stress reduced plant growth and photosynthetic pigments. Heatmap and principal component analysis (PCA) revealed that these toxic impacts were triggered by the greater oxidative damage and disruption of osmolyte homeostasis. However, pre-treatment of AT noticeably ameliorated the stress-induced toxicity as evident by enhanced chlorophyll, soluble protein, and soluble carbohydrate contents in AT-pretreated UVC-stressed plants relative to only stressed plants leading to the improvement of the plant growth and biomass. Moreover, AT pre-treatment enhanced endogenous AT and allantoate content, phenylalanine ammonia-lyase, non-enzymatic antioxidants, and the enzymatic antioxidants leading to reduced oxidative stress markers compared with only stressed plants, indicating the protective effect of AT against oxidative damage. Moreover, PCA displayed that the protective roles of AT strongly associate with the improved antioxidants. On the other hand, post-treatment of AT showed less efficacy in UVC stress mitigation relative to pre-treatment of AT. Overall, this finding illustrated that AT pre-treatment could be an effective way to counteract the UVC stress in tomato, and perhaps in other crop plants.

## 1. Introduction

Plants utilize sunlight as an energy source, which acts as an essential environmental signal to regulate growth and developmental processes via photosynthesis [1]. Higher plants’ photosynthetically active radiation is 400–700 nm [2]. Apart from this light range, plants are also exposed to numerous electromagnetic spectra, including ultraviolet (UV) radiation (UVC (100–280 nm), UVB (280–315 nm), and UVA (315–400 nm)) [3]. UVA radiation is not attenuated by ozone and is also not as detrimental as other UV radiations [4]. About 95% of UVB radiation is captivated by the ozone layer, whereas only 1 W m^−2^ UVB ray can reach the Earth’s surface [5,6]. On the other hand, the most energetic part of the UV spectrum is UVC, which hardly reaches the earth’s biosphere due to its filtration in the stratosphere’s ozone layer, but is used artificially to kill the pathogens [7,8]. This non-ionizing region of the electromagnetic spectrum regulates the morphological, physiological, and biochemical processes of plants as well as induces plant photomorphogenic development [9]. Moreover, a low dose of UV radiation can stimulate the plants’ antioxidant system, which may bolster the early adaptation of plants [10,11]. Despite the energetic driver of numerous plant responses, the damaging consequences of UV radiation for global plant productivity that are related to ozone depletion in the stratosphere are gradually surmounting and, therefore, regarded as stressors [12]. Studies showed that excessive UVB and UVC radiation could damage the DNA molecules by instigating dimerization and ionization of pyrimidines, resulting in disturbance of protein synthesis and disrupting their structures [1,6,8,13], leading to the drastic impairment in the normal cellular processes in plants [4,14]. Moreover, UV radiation with longer exposure time induced photosynthetic electron transport system inhibition. It also stimulates the production of excessive reactive oxygen species (ROS), which exceeds the antioxidant capacity of plants leading to membrane lipid and protein oxidation and DNA damage [1,3,15]. The mentioned concerning results were found mainly to photosynthetic tissues of plants as UV causes photosystem-II destruction [7,16]. In recent decades, UV-induced damage has augmented due to the depletion of the ozone layer by excess chlorofluorocarbons, which has a long half-life (50 to 150 years) and is being discharged excessively from the earth due to the anthropogenic causes [1,3]. Despite the filtration of natural UVC via the ozone layer, it has become a point of concern due to its future impact on natural ecosystems and agricultural productions due to atmospheric pollutant-induced ozone layer depletion [7,17,18].

Having said that, plant sensitivity to the radiation varies between plant species and varieties [19,20]. However, the plant possesses a plethora of defense strategies to combat the stressors, including UVC. Some fundamental mechanisms are: (i) regulation of compatible solutes such as proline (Pro), free amino acids (FAA), soluble protein, and sugar synthesis to compensate stress-induced osmotic imbalance [21]; (ii) stimulation of both non-enzymatic (e.g., glutathione (GSH), ascorbate (AsA), anthocyanin, flavonoids, α-tocopherol and total phenolic compounds (TPC)) and enzymatic (e.g., superoxide dismutase (SOD), catalase (CAT), ascorbate peroxidase (APX), soluble peroxidases (SPO), ionic peroxidases (IPO), and glutathione peroxidase (GPX)) antioxidant systems to detoxify ROS (e.g., superoxide anion (O_2_^•−^), hydrogen peroxide (H_2_O_2_), and hydroxyl radical (•OH)) for preventing ROS-induced lipid peroxidation and methylglyoxal (MG) production [22,23]; (iii) regulation of primary metabolites (e.g., organic acids, proteins, and carbohydrates) and secondary metabolites (e.g., allantoate, allantoin (AT) and others) and signaling molecules (hydrogen sulfide (H_2_S), nitric oxide (NO) and others) [24]; (iv) enhancement of photosynthetic pigment (chlorophyll (Chl) and carotenoid (Car)) synthesis to maintain optimum photosynthesis [25,26], and (v) regulation of some beneficial enzymes such as nitrate reductase (NR), glutathione *S*-transferase (GST), phenylalanine ammonia-lyase (PAL), and polyphenol oxidase (PPO) activity for plants [27,28,29].

Several strategies are being examined to upgrade the above-mentioned plant defense strategies against abiotic stress by the exogenous application of signaling molecules or stress tolerance-associated small compounds. However, the physiological function and significance of the accumulation of many stress-associated small compounds, especially purine metabolites, remain elusive. In plants, purines’ catabolism is a part of nitrogen metabolism; however, specific intermediate metabolites, such as AT, could also function in stress tolerance [30]. For example, reports in various plants describe the correlation between endogenous AT levels and protective physiological responses to both abiotic stresses such as nutrient deprivation [31,32], drought [33,34,35], salinity [36], and extended darkness [37] and biotic stress such as pathogen infection [38]. AT and its acyclic metabolites are collectively called ureides, and in tropical legumes, they serve as the vehicle for storage and xylem transport of symbiotically fixed nitrogen [39]. However, how AT reduces plant stress severity has been mostly uncharted to date. Gus’kov et al. [40] proposed AT as an antioxidant. Moreover, a study showed that exogenous AT efficiently reduced oxidative damage when applied exogenously to Arabidopsis seedlings [41]. Consistent with this result, genetic silencing of enzymes related to purine degradation decreased AT content in plants, leading to increased H_2_O_2_ in transgenic Arabidopsis plant [37,42]. However, no direct effect of AT was found on ROS homeostasis and constraining lipid peroxidation in vitro [43]. Thus, whether AT exerts a direct effect in vivo as an efficient antioxidant under UV stress conditions needs to be examined based on physiological and biochemical perspectives. Although the exogenous application of AT was examined in Arabidopsis under salt stress [41], nothing is known about the exogenous application of AT in tomato (*Solanum lycopersicum* L.) hybrid cultivar “F1-841” under UVC stress. However, it has been reported that exposure of tomato plants to enhanced UVC radiation levels can cause alterations in growth behavior [44]. However, the rationale behind this study was to evaluate whether certain purine metabolites, such as AT, participate in the mitigation of UVC stress in tomato plants. To test this hypothesis, we aimed to examine the role of exogenous AT in amelioration of the damaging impact on UVC stress based on several morphological, biochemical, and physiological attributes associated with (i) plant growth and biomass production, (ii) AT uptake, (iii) photosynthetic capacity, (iv) ROS metabolism and membrane damage, (v) enzymatic and non-enzymatic defense, (vi) MG detoxification, and (vii) accumulations of various osmolytes in tomato plants. 

## 2. Results

### 2.1. Doses of UVC and Exogenous AT

The phenotypic appearance and values of fresh and dry matter of shoot and root revealed that the UVC stress evoked a visible deleterious effect on tomato plants in a dose-dependent manner (Figure S1A and Table S1). The contents of AT and allantoate in tomato plants were found to be reduced dramatically when exposed to 0.6 W m^−2^ UVC radiation (Table S1). However, 0.3 W m^−2^ UVC exposure did not affect AT and allantoate contents of leaves in tomato plants (Table S1). Thus, we selected the first UVC irradiance (0.6 W m^−2^) that caused a significant reduction in the endogenous AT and allantoate contents to be used for applying different doses of exogenous AT. The UVC exposure dose 0.6 W m^−2^ and the sub-lethal dose 1.2 W m^−2^ were selected for further experiments in this study. 

The spraying of AT at different concentrations alleviated the damaging effect of UVC on tomato plants at different degrees (Figure S1B and Table S2). The best concentration of AT was 10^−4^ M or 100 nM, which alleviated the damaging impacts of UVC stress on tomato plants, as evident from the highest shoot fresh weight (SFW) (Table S2). The contents of AT and allantoate increased significantly as the concentration of AT application increased compared to only UVC-stressed tomato plants in a dose-dependent manner (Table S2). Overall, the concentration of 100 nM was the best concentration of AT that can alleviate the damaging effect of UVC stress and increased endogenous AT content at the highest level in the UVC-exposed tomato plants, and therefore, this concentration was selected as optimal and used for further experimentations (Table S2). 

### 2.2. AT Improved Growth, Water Relations, and Contents Photosynthetic Pigments

The optimal concentration of AT selected from the aforementioned experiment was applied at pre- and post-exposure to UVC to assess their efficacy in alleviating the stress incidence. The values of relative growth rate (RGR), SFW, root fresh weight (RFW), shoot dry weight (SDW), and root dry weight (RDW) showed a considerable decrease at “UVC1” and “UVC2” plants when compared with the growth parameter values of “C” tomato plants (Table 1). However, pre-treatment of AT improved RGR, SFW, RFW, SDW, and RDW at “AT+UVC1” and “AT+UVC2” plants, compared with their corresponding “UVC1” and “UVC2” plants, respectively (Table 1). Post-treatment of AT significantly increased SFW and excised leaf water retention (ELWR) at “UVC1+AT” plants and RDW at “UVC2+AT” plants but significantly decreased residual transpiration rate (RT) at “UVC2+AT” plants relative to that of the corresponding “UVC1” and “UVC2” plants, respectively (Table 1 and Table S3). 

Exposure to UVC hampered the photosynthetic pigments of tomato plants. “UVC1” and “UVC2” plants displayed significant reductions in Chl-*a*, Chl-*b*, Chl-*a+b*, and Car contents compared to that of “C” plants (Table 1). By contrast, pre-treatment of AT enhanced Chl-*a*, Chl-*b*, and Car contents in “AT+UVC1” and “AT+UVC2” plants in comparison with their corresponding “UVC1” and “UVC2” plants, respectively (Table 1). Post-treatment of AT did not significantly improve Chl-*a*, Chl-*b*, Chl-*a+b* contents except Car content at “UVC1+AT” plants but significantly increased Chl-*a*, Chl-*b*, Chl-*a+b* contents at “UVC2+AT” plants in comparison with the corresponding “UVC1” and “UVC2” plants, respectively (Table 1).

### 2.3. AT Differentially Maintained Water Content, Epicuticular Wax, Pro, FAA, Soluble Proteins, and Soluble Carbohydrates

**Figure 1:**
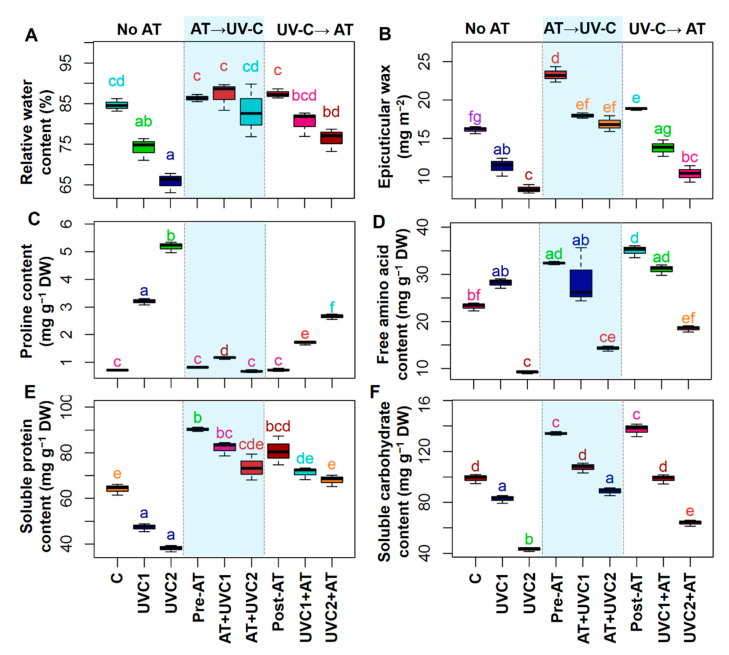
Effects of pre-treatment and post-treatment of allantoin (AT) on the relative water (RWC) content (**A**), epicuticular wax (**B**), proline (Pro) content (**C**), free amino acid (FAA) content (**D**), soluble protein (SP) content (**E**), and soluble carbohydrate (SC) content (**F**) in tomato plants grown 15 days in presence and absence of UVC stress. Each boxplot shows values of five independent replicates (*n* = 5). Boxplots specified with the same letter(s) are statistically non-significant at *p* < 0.05 based on Tukey’s test. “C”, no AT and grown under non-stress condition; “UVC1”, exposed to 0.6 W m^−2^ UVC irradiation; “UVC2”, exposed to 1.2 W m^−2^ UVC irradiation; “Pre-AT”, pretreated with 100 nM AT and thereafter exposed to 0 W m^−2^ UVC irradiation; “AT+UVC1”, pretreated with 100 nM AT and after that exposed to 0.6 W m^−2^ UVC irradiation; “AT+UVC2”, pretreated with 100 nM AT and after that exposed to 1.2 W m^−2^ UVC irradiation; “Post-AT”, exposed to 0 W m^−2^ UVC irradiation and thereafter treated with 100 nM AT; “UVC1+AT”, exposed to 0.6 W m^−2^ UVC irradiation and thereafter treated with 100 nM AT; “UVC2+AT”, exposed to 1.2 W m^−2^ UVC irradiation and after that treated with 100 nM AT.

Exposure of tomato plants to UVC stress displayed a significant decrease in the relative water content (RWC), epicuticular wax, soluble proteins (SP), and soluble carbohydrates (SC), while augmentation of the level of Pro in the leaves of “UVC1” and “UVC2” plants, respectively, and decreased FAA content in “UVC2” plants, when compared with that of “C” plants (Figure 1). Pre-treatment of AT significantly increased RWC, epicuticular wax, SP content, and SC content but decreased Pro content in the leaves of “AT+UVC1” and “AT+UVC2” plants, respectively, compared to that found in the corresponding “UVC1” and “UVC2” plants (Figure 1A–C,E,F). Post-treatment of AT significantly improved SP content and SC content but declined Pro content, and no significant change in epicuticular wax and FAA content in the leaves of “UVC1+AT” and “UVC2+AT” plants, respectively, compared to that found in the corresponding “UVC1” and “UVC2” plants was found (Figure 1). Furthermore, post-treatment of AT significantly increased RWC in “UVC2+AT” plants, whereas no significant change in “UVC1+AT” plants compared with their corresponding “UVC1” and “UVC2” plants was found (Figure 1A).

### 2.4. AT Attenuated Oxidative Damage

**Figure 2:**
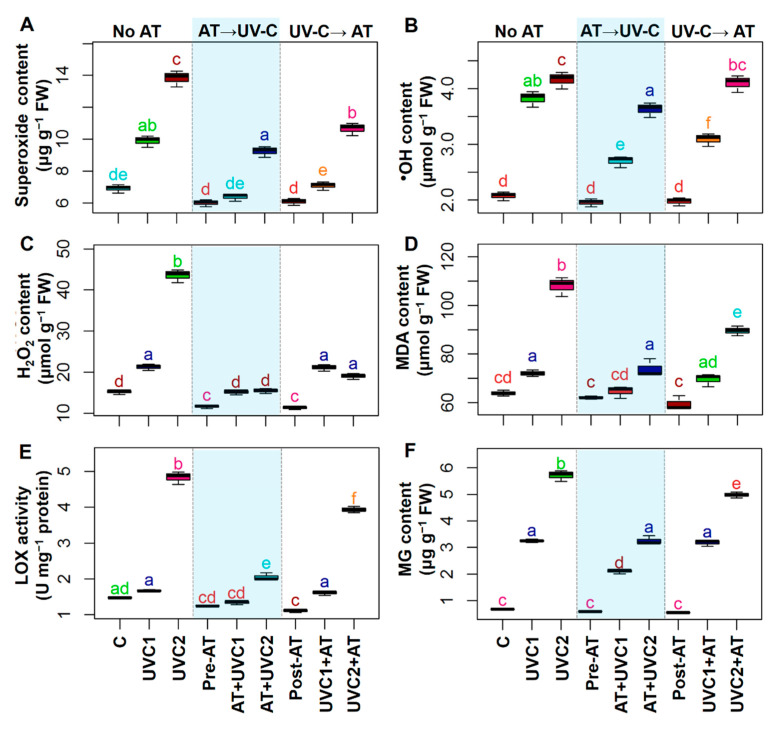
Effects of pretreatment and post-treatment of allantoin (AT) on the superoxide anion (O_2_^•−^) content (**A**), hydroxyl radical (^•^OH) content (**B**), hydrogen peroxide (H_2_O_2_) content (**C**), malondialdehyde (MDA) content (**D**), lipoxygenase activity (LOX) (**E**), and methylglyoxal (MG) content (**F**) in tomato plants grown 15 days in presence and absence of UVC stress. Each boxplot shows values of five independent replicates (*n* = 5). Boxplots specified with the same letter(s) are statistically non-significant at *p* < 0.05 based on Tukey’s test. Treatments are the same as Figure 1.

Exposure of tomato plants to UVC stress displayed a sharp increase in O_2_^•−^, ^•^OH, H_2_O_2_, malondialdehyde (MDA), and MG in both “UVC1” and “UVC2” plants and lipoxygenase (LOX) activity in “UVC2” plants (Figure 2) compared with that of “C” plants. Pre-treatment of AT significantly decreased O_2_^•−^, ^•^OH, H_2_O_2_, MDA, LOX activity, and MG content in the leaves of “AT+UVC1” and “AT+UVC2” plants, respectively, compared to that found in the corresponding “UVC1” and “UVC2” plants (Figure 2). Post-treatment of AT significantly reduced O_2_^•−^ and ^•^OH in “UVC1+AT” plants and H_2_O_2_, MDA, LOX, and MG in “UVC2+AT” plants compared to that found in the corresponding “UVC1” and “UVC2” plants (Figure 2).

### 2.5. AT Improved Non-Enzymatic Antioxidant Status and Secondary Metabolites

**Figure 3:**
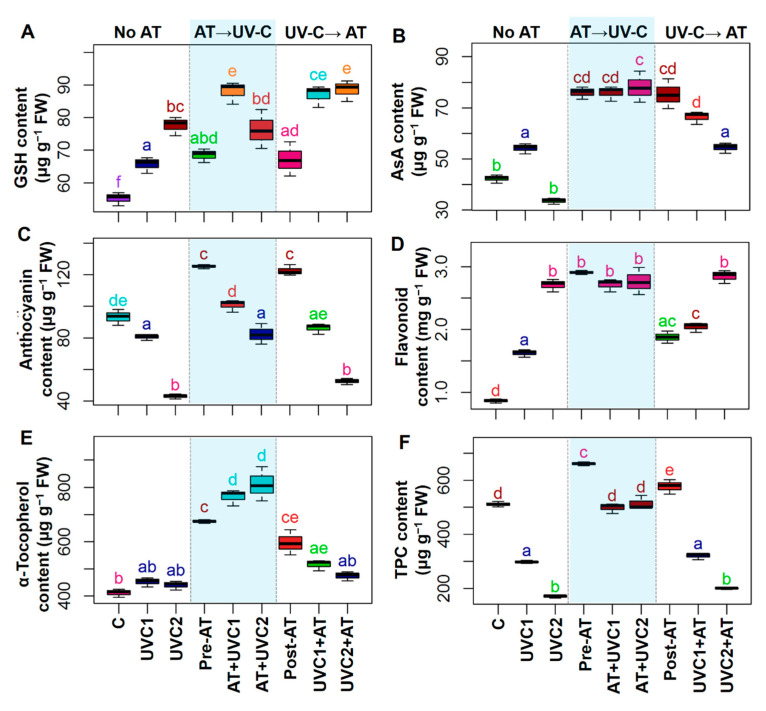
Effects of pre-treatment and post-treatment of allantoin (AT) on glutathione (GSH) (**A**), ascorbic acid (AsA) (**B**), anthocyanin (**C**), flavonoid (**D**), α-tocopherol (**E**), and phenolic compounds (TPC) contents (**F**) in tomato plants grown 15 days in presence and absence of UVC stress. Each boxplot shows values of five independent replicates (*n* = 5). Boxplots specified with the same letter(s) are statistically non-significant at *p* < 0.05 based on Tukey’s test. Treatments are the same as Figure 1.

Exposure of tomato plants to UVC stress displayed a sharp increase in GSH content and flavonoid content but showed a significant decrease in anthocyanin content and TPC in “UVC1” and “UVC2” plants relative to that observed in “C” plants (Figure 3A,C–F). Total AsA contents in “UVC1” plants significantly increased, whereas α-tocopherol content displayed a non-significant change in “UVC1” and “UVC2” plants relative to that observed in “C” plants (Figure 3B,E). Pre-treatment of AT significantly increased GSH content and flavonoid content in “AT+UVC1” plants but increased AsA, anthocyanin, α- tocopherol, and TPC contents in “AT+UVC1” and “AT+UVC2” plants compared to that found in the corresponding “UVC1” and “UVC2” plants (Figure 3). Post-treatment of AT also significantly improved GSH and AsA content in “UVC1+AT” and “UVC2+AT” plants and flavonoid content in “UVC1+AT” plants compared to that found in the corresponding “UVC1” and “UVC2” plants (Figure 3A,B,D).

### 2.6. AT Improved Different Enzymatic Activities

**Figure 4:**
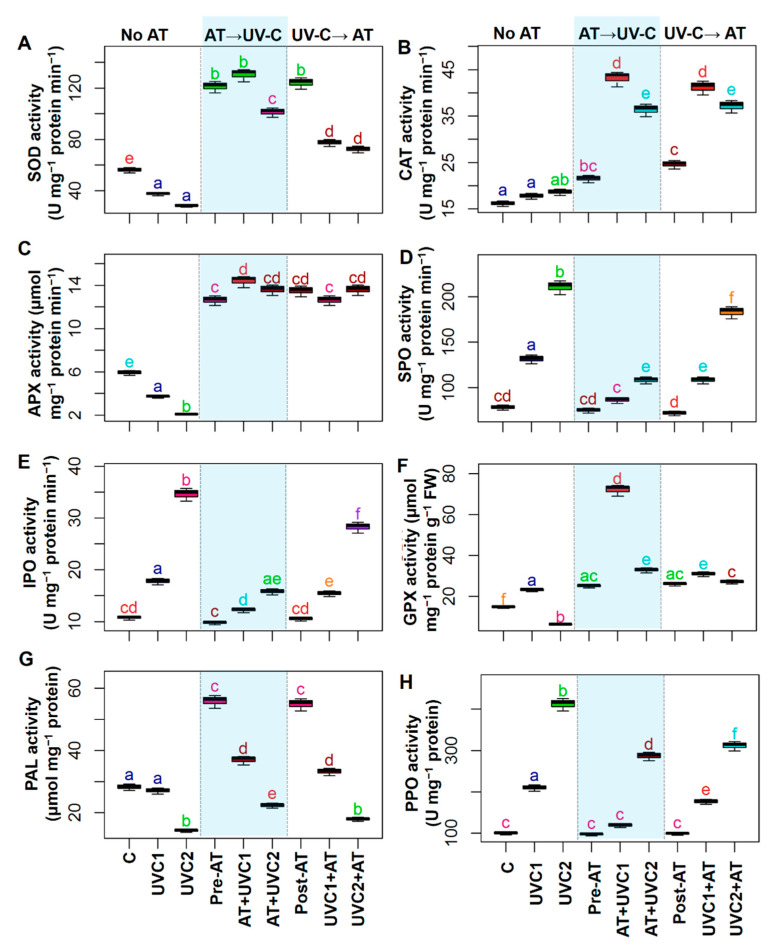
Effects of pre-treatment and post-treatment of allantoin (AT) on superoxide dismutase (SOD) activity (**A**), catalase (CAT) activity (**B**), ascorbate peroxidase (APX) activity (**C**), soluble peroxidases (SPO) activity (**D**), ionic peroxidases (IPO) activity (**E**), glutathione peroxidase (GPX) activity (**F**), phenylalanine ammonia-lyase (PAL) contents (**G**), and polyphenol oxidase (PPO) activity (PPO) (**H**) in tomato plants grown 15 days in presence and absence of UVC stress. Each boxplot shows values of five independent replicates (*n* = 5). Boxplots specified with the same letter(s) are statistically non-significant at *p* < 0.05 based on Tukey’s test. Treatments are the same as Figure 1.

In comparison with “C” plants, “UVC1” and “UVC2” plants displayed a significant decrease in the activities of SOD and APX, but showed a non-significant change of CAT activities (Figure 4A–C). On the other hand, in comparison with respective “UVC1” and “UVC2” plants, pretreated “AT+UVC1” and “AT+UVC2” plants showed significantly enhanced activities of SOD, CAT, and APX (Figure 4A–C). A similar increment of SOD, CAT, and APX activities was also found in post-treated “UVC1+AT” and “UVC2+AT” plants in comparison with their corresponding “UVC1” and “UVC2” plants, respectively (Figure 4A–C). Both SPO and IPO activities were sharply increased in “UVC1” and “UVC2” plants in comparison with that of “C” plants (Figure 4D,E). On the other hand, both pre- and post-AT treatment in stressed plants showed a decline in SPO and IPO activities in comparison with their respective only stressed plants (Figure 4D,E). GPX activity increased in “UVC1” plants, but it dropped in “UVC2” plants versus that of “C” plants (Figure 4F). In contrast, both pre- and post-treatment of AT in stressed plants showed an increment of GPX activities versus their respective only stressed plants (Figure 4F). PAL activity displayed a sharp decline in “UVC2” plants compared to “C” plants (Figure 4G). On the other hand, pre-treatment of AT increased PAL activity both in “AT+UVC1” and “AT+UVC2” plants, whereas post-treatment of AT increased it in “UVC1+AT” plants compared to that found in the corresponding “UVC1” and “UVC2” plants (Figure 4G). PPO activity significantly increased in “UVC1” and “UVC2” plants compared to “C” plants. Pre- and post-treatment of AT significantly decreased PPO activity in “UVC1+AT” and “UVC2+AT” in comparison with respective “UVC1” and “UVC2” plants (Figure 4H). 

### 2.7. AT Regulates Endogenous Metabolites, Signaling Molecules, GST, and PPO Activity

**Figure 5:**
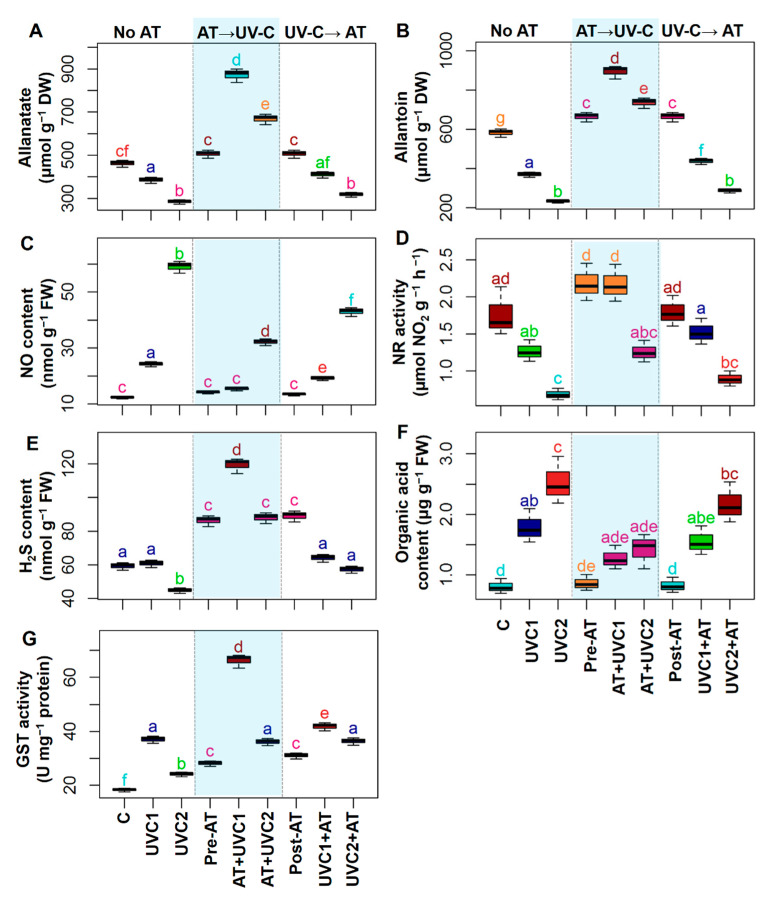
Effects of pretreatment and post-treatment of allantoin (AT) on allantoate contents (**A**), endogenous AT content (**B**), nitric oxide (NO) content (**C**), nitrate reductase (NR) activity (**D**), hydrogen sulfide (H_2_S) content (**E**), organic acid content (**F**), and glutathione *S*-transferase (GST) activity (**G**) in tomato plants grown 15 days in presence and absence of UVC stress. Each boxplot shows values of five independent replicates (*n* = 5). Boxplots specified with the same letter(s) are statistically non-significant at *p* < 0.05 based on Tukey’s test. Treatments are the same as Figure 1.

In comparison with “C” plants, “UVC1” and “UVC2” plants showed significant decreases in allantoate and endogenous AT contents, whereas they displayed a significant increase in NO content, organic acid content, and GST content (Figure 5A–C,F,G). Then, “UVC2” plants showed a significant decreased in NR activity and H_2_S content compared with “C” plants (Figure 5D,E). Pre-treatment of AT significantly increased allantoate, endogenous AT, H_2_S, and GST contents but decreased NO content in “AT +UVC1” and “AT+UVC2” plants, respectively, in comparison with respective “UVC1” and “UVC2” plants (Figure 5 A–C,E,G). Pre-treatment of AT also improved NR activity in “AT +UVC1” plants but declined organic acid content in “AT+UVC2” plants compared with respective “UVC1” and “UVC2” plants (Figure 5D,F). Post-treatment of AT significantly increased GST activity and decreased NO content in “UVC1+AT” and “UVC2+AT” and increased considerably endogenous AT content in “UVC1+AT” plants and H_2_S content in “UVC2+AT” in comparison with respective “UVC1” and “UVC2” plants (Figure 5B,C,G).

### 2.8. Treatment–Variable and Variable–Variable Interaction 

**Figure 6:**
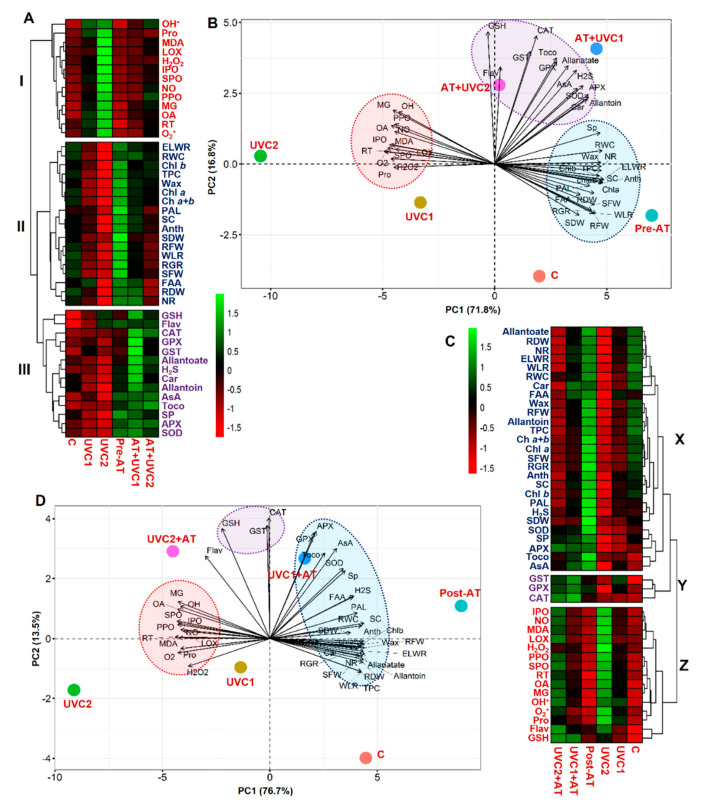
Hierarchical clustering with heatmap (**A**,**C**) and principal component analysis (PCA) (**B**,**D**) show the treatment-variable relationships. In hierarchical clustering and heatmap, the mean values of various parameters obtained in this study were normalized and analyzed. Three distinct clusters (cluster I–III for pre-treatment (**A**) and cluster X-Z for post-treatment (**C**)) were identified at the variable level. In the heatmap, the color scale displays the intensity of normalized mean values of different parameters. The entire dataset was also divided to analyze PCA of AT pre-treatment (**B**) and post-treatment (**D**). The variables included RGR, relative growth rate; SFW, shoot fresh weight; RFW, root fresh weight; SDW, shoot dry weight; RDW, root dry weight; WLR, water loss rate; ELWR, excised leaf water retention; RT, residual transpiration; Chl, chlorophyll; Car, carotenoid; RWC, relative water content; Wax, epicuticular wax; Pro, proline content; FAA, free amino acid content, SP, soluble protein content; SC, soluble carbohydrate content; O_2_^•−^, superoxide content; ^•^OH, hydroxyl radical content; H_2_O_2_, hydrogen peroxide content; MDA, malondialdehyde content; LOX, lipoxygenase activity; MG, methylglyoxal content; GSH, glutathione content; AsA; ascorbic acid content; Anth, anthocyanin content; Flav, flavonoid content; Toco, α-tocopherol content; TPC, total phenolic compound content; SOD, superoxide dismutase activity; CAT, catalase activity; APX, ascorbate peroxidase activity; SPO, soluble peroxidases activity; IPO, ionic peroxidases activity; GPX, glutathione peroxidase activity; PAL, phenylalanine ammonia-lyase activity; allantoate content, endogenous allantoin content; NO, nitric oxide content; NR, nitrate reductase activity; H_2_S, hydrogen sulfide content; OA, organic acid content; GST, glutathione S-transferase; and PPO, polyphenol oxidase. Treatments are the same as Figure 1.

The mean values of all morpho-physiological and biochemical parameters were used to perform a heatmap with hierarchical clustering and PCA, which were undertaken both for AT pre- and post-treatments separately. Hierarchical clustering for AT pre-treatment revealed the classification of all studied parameters divided into three clusters (cluster-Ι–ΙΙΙ) (Figure 6A). Cluster-Ι encompassed O_2_^•−^, ^•^OH, H_2_O_2_, MDA, LOX, MG, PPO, IPO, SPO, RT, NO, Pro, and OA of plants. Cluster-Ι parameters showed an increasing pattern in “UVC1” and “UVC2” tomato plants but displayed a decreasing trend in “AT+UVC1” and “AT+UVC2” plants. Variables SDW, RFW, SFW, RGR, RDW, ELWR, RWC, wax (epicuticular wax), WLR (water loss rate), Chl-*a*, Chl-*b*, Chl-*a+b*, TPC, PAL, NR, SC, Anth (anthocyanin), and FAA were grouped in the cluster-ΙΙ. Compared with “C” plants, cluster-ΙΙ parameters displayed a decreasing pattern in “UVC1” and “UVC2” tomato plants but showed an increasing pattern in “AT+UVC1” and “AT+UVC2” plants (Figure 6A). GSH, Flav (flavonoid), CAT, GPX, GST, H_2_S, Car, allantoin, allantoate, AsA, Toco (α-tocopherol), SP, APX, and SOD of leaves were grouped in cluster-ΙΙΙ. Compared with “C” plants, cluster-ΙΙΙ parameters displayed a decreasing pattern in “UVC1” and “UVC2” tomato plants but showed an increasing pattern in “AT+UVC1” and “AT+UVC2” plants (Figure 6A). Furthermore, a PCA biplot was performed to determine the degree of association within the treatments and variables. The first two PCA components, PC1 and PC2, combinedly explained 88.6% of the data variability (Figure 6B). PCA delineated that variables of cluster-Ι were strongly connected with “UVC1” and “UVC2” plants. However, cluster-ΙΙΙ variables were more strongly associated with “AT+UVC1” and “AT+UVC2” plants. Moreover, cluster-ΙΙ variables were strongly associated with AT pretreated “Pre-AT” plants (Figure 6B).

In terms of post-treatment of AT, cluster-X of heatmap contained RGR, SFW, RFW, SDW, RDW, Chl-*a*, Chl-*b*, Chl-*a+b*, Car, ELWR, WLR, RWC, and epicuticular wax; SOD, APX, and PAL; allantoin, allantoate, Anth (anthocyanin), SP, SC, Toco (α-tocopherol), NR, FAA, TPC, H_2_S, and AsA. Compared with “C” plants, Cluster-X parameters showed a decreasing pattern in “UVC1” and “UVC2” tomato plants but showed an increasing pattern in “UVC1+AT” and “UVC2+AT” plants (Figure 6C). GST, GPX, and CAT were grouped in Cluster-Y. On the other hand, IPO, NO, MDA, LOX, PPO, SPO, RT, OA, MG, ^•^OH, H_2_O_2_, O_2_^•−^, Pro, Flav (flavonoid), and GSH were grouped in Cluster-Z. Compared with “C” plants, Cluster-Z parameters showed an increasing pattern in “UVC1” and “UVC2” tomato plants but showed a decreasing pattern in “UVC1+AT” and “UVC2+AT” plants (Figure 6C). The first two PCA components, PC1 and PC2, combinedly explained 90.2% of the data variability (Figure 6D). PCA delineated that variables of cluster-X were strongly connected with “UVC1+AT” and AT post-treated “post-AT” plants. However, Cluster-Z variables were more strongly associated with “UVC1” and “UVC2” plants. Moreover, cluster-Y variables are related to “UVC1+AT” and “UVC2+AT” (Figure 6D).

## 3. Discussion

Exposure to UVC causes an impediment to plant growth progression due to its adversative effects on physiological and biochemical processes [1,6,45]. However, exogenous AT might be used to improve UVC stress tolerance due to its ability to regulate plant tolerance to several abiotic stresses such as nutrient deprivation, drought, and salinity [31,32,33,34,35,36,41]. In the present experiment, for the first time, we used AT exogenously for UV stress alleviation, and overall, we demonstrated that exogenous foliar application of AT, particularly pre-treatment of AT, could reduce the harmful consequences of UVC stress on the tomato plant.

Plant growth and biomass production are highly vulnerable to UVC stress (Table 1), as previously observed in several plant species, including pepper (*Capsicum annuum*) under UVC stress [45] and cotton (*Gossypium hirsutum*) under UVB stress [46]. In the current study, UVC stress was manifested in terms of curtailed RGR, SFW, RFW, SDW, and RDW in “UVC1” and “UVC2” tomato plants (Table 1), implying that exposure to UVC caused phenotypic abnormalities and a reduction in the growth of tomato plants through interfering with various physiological and metabolic processes of plants. Furthermore, the growth of a plant is closely related to its photosynthetic efficacy [25,26], sufficient absorption of water [47], and ROS management via antioxidant activity [22,48], and all of those aforementioned processes were rigorously affected by UVC stress in this study (Figure 1, Figure 2, Figure 3 and Figure 4), resulting in reduced growth performance of tomato plants (Table 1). On the other hand, AT pre-treatment improved the growth and biomass production of “AT+UVC1” and “AT+UVC2” tomato plants (Table 1) as also observed in Arabidopsis subjected to salt stress [41]. This is pointing towards the rise of the NH_4_^+^ released via AT catabolism contributed to the nitrogen homeostasis in bread wheat (*Triticum aestivum*) plants [49], thereby supporting the alleviation of the UVC-induced damage on tomato plants. Importantly, AT post-treatment in “UVC1+AT” and “UVC2+AT” plants also improved growth parameters of tomato plants but not as effectively as pre-treatment of AT (Table 1), which is validated by the stronger positive association of “AT+UVC1” and “AT+UVC2” with growth-related parameters than that of “UVC1+AT” and “UVC2+AT” tomato plants, respectively, in the PCA (Figure 6B,D).

The photosynthetic rate determines the overall growth performance of plants, which depends directly on capturing light energy by Chls, particularly Chl-*a* and Chl-*b* and Car [25,26,50]. In the present study, Chl-*a* and Chl-*b* contents in tomato leaves were significantly declined in “UVC1” and “UVC2” plants, demonstrating that reduced Chls and Car contents might hamper the photosynthesis in tomato plants as reflected by the reduced plant growth and biomass (Table 1). The reduction in photosynthetic pigments might be because of the ROS-induced disorganization of thylakoid membranes [26], and lack of sufficient mineral nutrient uptake [51] might be due to the improper translocation of water as evidenced by reduced RWC, ELWR, and WLR (Figure 1A and Table S3). However, plant photosynthetic efficiency directly regulates the levels of primary metabolites such as sugars and proteins in plant tissues, particularly under abiotic stress conditions [52,53]. In the current study, we also observed a positive relationship between Chl and Car contents with SP, SC, and FAA contents in the “AT+UVC1” and “AT+UVC2” plants (Figure 1D–F and Table 1), as also confirmed by a strong positive correlation of Chls and Car with SP, SC, and FAA in the PCA (Figure 6B). These results suggested that AT strengthened the tomato plant’s photosynthetic efficiency and maintained higher sugars and proteins levels, which might help tomato plants to combat stress-induced toxicity by providing energy and metabolites for numerous biochemical pathways.

Water scarcity at cellular levels is a common phenomenon in abiotic stress [54,55,56], as also observed in “UVC1” and “UVC2” plants, evidenced by reduced RWC causing poor growth performance (Figure 1A). A similar result was also observed in dermaneh (*Artemisia sieberi*) plants under heat stress [57] and potato (*Solanum tuberosum*) plants under drought stress [58]. This reduction in water content caused might be due to the less accumulation of epicuticular wax on leaf surface, leading to an increase in RT (Figure 1A,B and Table S3). Therefore, over time, due to the physiological water scarcity, tomato plants might attempt to lose as little water as possible as evidenced by reduced WLR in tomato plants after exposure to UVC stress for 15 days (Table S3). Thus, the tomato plants tended to roll their leaf margins in response to UVC stress (Figure S2). However, to retain optimum water content, tomato plants also tend to accrue organic solutes such as Pro and organic acids [59], as also noticed in this study (Figure 1A,C and Figure 5F). These results demonstrated that UVC-stressed tomato plants responded to increased water loss by triggering Pro and organic acid accumulation (Figure 1C and Figure 5F) as also observed in dermaneh plants under heat stress [57] and potato plants under drought stress [58]. These findings lead us to conclude that UVC stress negatively impacts plant water status and water translocation in tomato plants, which might contribute to the poor nutrient absorption and stomatal function, resulting in the tomato plant growth and biomass reduction. On the other hand, pre-treatment of AT improved the water status of “AT+UVC1” and “AT+UVC2” tomato plants (Figure 1A), signifying a crucial role of AT in dealing with cellular dehydration in tomato plants, resulting in no need for excessive Pro accumulation (Figure 1C). AT also increased the epicuticular wax accumulation on the leaf surface of UVC-stressed plants; thus, AT-treated plants showed little leaflets rolling compared to only UVC-stressed plants (Figure S3). PCA analysis revealed that pre-treatments of AT effectively improved water relation parameters under low and high UVC stress, whereas post-treatments of AT were only effective under low UVC stress (Figure 6B,D).

The accumulation of ROS, which is triggered by UV stress, is thought to be associated with photo-oxidative damage [1], and was manifested in UVC-stressed plants in a concentration-dependent manner (Figure 2A–C). Our results demonstrated that “AT+UVC1” and “AT+UVC2” plants accumulated less ROS, such as O_2_^•−^, ^•^OH, and H_2_O_2_, and subsequently reduced LOX activity, MDA, and MG contents (Figure 2), signifying the ameliorative role of AT against ROS-induced oxidative burden and in the protection of plasma membrane integrity. Following these findings, enzymatic antioxidant assays confirmed that “AT+UVC1” and “AT+UVC2” tomato plants exhibited enhanced activities of SOD, CAT, APX, GPX, and GST, compared with UVC-stressed tomato plants (Figure 4A–C,F). It is plausible that the boosted activity of SOD in the leaves of AT-pre-treated plants might imply the attenuation of O_2_^•−^ levels (Figure 2A and Figure 4A) by promoting the dismutation of O_2_^•−^ into H_2_O_2_ [60]. Subsequently, it is likely that the toxic H_2_O_2_ was then eventually eliminated in AT-pre-treated tomato plants as a result of the increased activities of CAT and APX (Figure 2C and Figure 4B,C). In addition, the greater activities of GPX and GST in the leaves of AT-pre-treated tomato plants (Figure 4F and Figure 5G) indicated activation of GSH-dependent peroxide scavenging systems to provide enhanced protection against the UVC-induced toxic byproducts [61]. Intriguingly, unlike other enzymatic antioxidant activities, SPO and IPO activity was reduced upon AT supplementation in “AT+UVC1” and “AT+UVC2” plants (Figure 4D,E). The reasons behind the decrease in peroxidases in response to AT are not well-understood yet. Therefore, in a future study, an examination of the expression of the whole SPO and IPO biosynthesis-related gene family in AT-treated tomato plants under UVC conditions is warranted and will provide additional clarity regarding the molecular nature of the AT-induced responses. Apart from enzymatic antioxidants, our results also showed that the AT-pre-treated plants accumulated higher levels of non-enzymatic antioxidants (e.g., AsA, anthocyanin, α-tocopherol, and TPC) relative to only “UVC1” and “UVC2” tomato plants, which also contributed to the maintenance of proper ROS homeostasis under UVC stress conditions (Figure 3B,C,E,F). Anthocyanin, α-tocopherol, flavonoid, and TPC are well-known secondary metabolites that play a crucial role in protecting cell membranes from oxidative damage by scavenging free radicals under abiotic stress, including UV stress [62]. Importantly, PAL activity, which is related to the biosynthesis of phenolic compounds and found at the interface between primary and secondary metabolism [63], was decreased in UVC-stressed tomato plants (Figure 4G), as also observed in *Lotus japonicas* under salt stress [64]. The reduced PAL and increased PPO activity explain the reduction in TPC in the current study (Figure 3F and Figure 4G,H). On the contrary, pre-treatment of AT improved PAL activity along with anthocyanin, α-tocopherol, and TPC in “AT+UVC1” and “AT+UVC2” tomato plants (Figure 3C,E,F). Furthermore, flavonoids and GSH content showed higher accumulation in UVC-stressed plants (Figure 3A,D), as also observed in combined O_3_ and UV radiation in soybean [65]. On the other hand, “AT+UVC1” tomato plants showed significantly enhanced GSH and flavonoid contents, whereas “AT+UVC2” tomato plants displayed a non-significant change compared to their respective only stressed tomato plants (Figure 3A,D). Our PCA analysis displayed that enzymatic antioxidants and non-enzymatic antioxidants showed a positive relation, and ROS and membrane destruction showed a negative relation in “AT+UVC1”, “AT+UVC2”, and “UVC1+AT” plants, whereas “UVC2+AT” plants showed low association (Figure 6B,D). This PCA result concludes that pre-treatment of AT effectively improves plant antioxidant parameters, subsequently reducing ROS and membrane destruction both under low and high UVC stress conditions, but post-treatment of AT is only effective under low UVC stress conditions.

Plant synthesized many metabolites and signaling molecules such as allantoate, AT, organic acids, NO, and H_2_S. associated with normal plant growth and developmental processes [24,35]. Allantoate and AT, urides, nitrogen storage, and transport compounds that recover nitrogen from purine rings showed substantial promise in plant stress tolerance. Endogenous accumulation of allantoate and AT in response to abiotic stress showed tolerance to a wide range of stresses [33,66,67,68]. In our experiment, both allantoate and endogenous AT decreased in UVC-stressed tomato plants (Figure 5A,B), which might interrupt the uride degradation in plants that is responsible for maintaining proper nitrogen homeostasis [69], resulting in decreased tomato plant protection against UVC stress (Table 1).

In plants, some signaling molecules such as NO possesses a dual role, both positive and negative, during abiotic stress [70]. Recent evidence suggests that exogenous application of SNP, a NO donor, protected *Lactuca sativa* seedlings from UVB stress by upregulating antioxidant properties [71]. On the other hand, excess NO accumulation could also assist the ROS-induced programmed cell death of plant cells [72]. These pieces of evidence suggest that the role of NO depends on its concentration. In the current experiment, NO content increased in UVC-stressed plants (Figure 5C) as also as in drought-stressed maize (*Zea mays*) and trifoliate orange (*Poncirus trifoliata*) [73,74]. This increased NO in combination with increased ROS (Figure 2A–C and Figure 5C) might produce peroxynitrite that could induce a hypersensitive response, a type of plant programmed cell death and necrosis [72], leading to suppressed tomato plant phenotype (Table 1). On the contrary, “AT+UVC1” and “AT+UVC2” tomato plants showed a reduced NO content (Figure 5C), indicating AT-mediated nitrosative stress mitigation in tomato plants. In searching for NO sources in UVC stress, we measured NR activity, which decreased while NO content increased in UVC-stressed plants (Figure 5C,D), and NO content and NR activity exhibited a strong negative correlation in PCA. This result leads us to presume that the origin of NO in UVC-stressed tomato plants was from a non-enzymatic source or oxidative pathway that can lead to efficient and rapid production of NO from nitrite or other sources [75]. Previous studies showed that higher endogenous H_2_S improves abiotic stress tolerance in various plant species [76,77]. In the current study, in “UVC2” plants, H_2_S content decreased significantly (Figure 5E), resulting in an imbalance of the *S*-sulfhydration process, vital for functional changes of protein activities [78]. However, exogenous application of AT increased the H_2_S content in “AT+UVC1” and “AT+UVC2” plants (Figure 5E), which might positively regulate the plant physiological processes against UVC-stressed tomato plants [79]. Overall, the PCA clearly showed that “AT+UVC1” and “AT+UVC2” plants showed a stronger association with H_2_S content, endogenous AT, and allantoate contents than that of “UVC1+AT” and “UVC2+AT” plants. This result indicates that AT pre-treatment-mediated UVC stress tolerance mainly because of the upregulation of levels of H_2_S, endogenous AT, and allantoate and post-treatment of AT failed to do that and, therefore, showed reduced UVC stress tolerance.

From the above discussion, we found that pre-treatment of AT is more effective than post-treatment of AT in UVC-stressed tomato plants. Several factors might be related to this result. Firstly, it is well understood that UVC stress causes genotoxic harm in the plants by destruction of the genetic material and macromolecules of plants. Moreover, UV photons absorbed by DNA can result in the formation of cyclobutane pyrimidine dimers and some other compound which cause DNA modification, resulting in the inhibition of transcription and translation process [13,80]. Therefore, when UVC had already altered the genetic material of the plants, it was difficult to obtain the post-AT treated improvement of tomato plants. Secondly, as already mentioned, plant’s enzymatic antioxidant, non-enzymatic antioxidant, and secondary metabolites are crucial for plant protection against abiotic stress-induced ROS [62]. Especially flavonoids act as sunscreen in plants that shield the inner cells of the epidermis from harmful radiations [81,82]. Interestingly, in our experiment, secondary metabolites and non-enzymatic antioxidants such as GSH, AsA, anthocyanin, flavonoid, α-tocopherol, and TPC, and enzymatic antioxidants such as SOD and APX and PAL activity were higher in AT treated non-stressed tomato plants (Figure 3 and Figure 4A,C,G). This result was further validated by PCA analysis of our data (Figure 6B,D). It was found that pre-treatment of AT to “AT+UVC1” and “AT+UVC2” are strongly associated with improved plant antioxidant parameters compared with post-treatment of AT in “UVC1+AT” plants. Moreover, post-treatment of AT in “AT+UVC2” plants showed no association with antioxidant parameters (Figure 6D). These results support our assumption that pre-treatment of AT provoked the antioxidant system of the plant along with accumulating UVC absorbing compounds in tomato plants that quickly responded against UVC stress resulting in better protection in tomato plants.

## 4. Materials and Methods

### 4.1. Experimental Conditions, Crop, and UVC Stress

A pot experiment was carried out in the glasshouse under natural conditions of humidity (67–75%), temperature (maximum 30 and 15 °C), and light. Tomato (Solanum lycopersicum L.) hybrid F1–841 was used as a study crop. Healthy seeds of the tomato were surface sterilized with 0.1% mercuric chloride for 5 min and then rinsed vigorously with sterile distilled water (dH_2_O) three times. The seeds were cultivated in trays filled with 100% Peat moss and kept at 25 °C. The trays received 150 mg/L water-soluble fertilizer. The seedlings (35 days) were then transplanted to pots containing 1 Kg clay soil (loamy-clay-sandy, pH 7.3, electrical conductivity 1.10 dSm^−1^, organic matter 1.3 mg Kg^−1^, TSS 0.843, Na^+^ 7.6 mg Kg^−1^, K^+^ 0.35 mg Kg^−1^, Ca^2+^ 0.43 mg Kg^−1^, and Mg^2+^ 0.054 mg Kg^−1^). Three seedlings of similar lengths and sizes were transplanted per pot to be used throughout the experiments, and the pots were fertilized by 50% nutrient solution [83]. The pots were irrigated with Hoagland’s solution (100 mL) once a week. The pots were irrigated daily with the calculated amount of water to maintain soil moisture content at the field capacity.

Tomato seedlings were subjected to UVC stress using the UVC radiation-emitting lamp (Model UVC TL, Philips, 20W). The applied doses were obtained by varying the exposure time to the set distance, using the equation of López et al. [84]: D = (F×t)/1000, (1) where D is the dose of the radiation applied (kJ m^−2^), F is the radiant flow (W m^−2^), and t is the time of exposure (in seconds). The plants were placed at a 40 cm distance from the UVC emitting lamp. The UVC lamps were stabilized by turning on lamps at 11:00 a.m. for the calculated time of each irradiance treatment and then closed; this process was conducted for three days.

### 4.2. Evaluation of the Effect of the Optimal Dose of AT on UVC Stress

The tomato plants (40 days seedlings sub-cultured in 1 kg clay soil) were divided into three groups— group I: the plants were sprayed with the distilled water and acted as negative control and experienced UVC stress at doses 0, 0.6, and 1.2 W m^−2^; group II: the best concentration of AT (10^−4^ M or 100 nM; this concentration was optimized from the previous experiment; Tables S1 and S2) was applied 1 day before UVC irradiance at doses 0, 0.6, and 1.2 W m^−2^; and group III: the plants were subjected firstly to UVC irradiation at doses 0, 0.6, and 1.2 W m^−2^ and after 1 day sprayed with the best dose of AT. Five pots were used per treatment, and three plants per pot were maintained in Randomized Complete Block Design. Thus, our experiment consisted of a total of nine treatments as follows: (i) C, no AT and grown under non-stress condition; (ii) UVC1, exposed to 0.6 W m^−2^ UVC irradiation; (iii) UVC2, exposed to 1.2 W m^−2^ UVC irradiation; (iv) Pre-AT, pretreated with 100 nM AT and after that exposed to 0 W m^−2^ UVC irradiation; (v) AT+UVC1, pretreated with 100 nM AT and after that exposed to 0.6 W m^−2^ UVC irradiation; (vi) AT+UVC2, pretreated with 100 nM AT and after that exposed to 1.2 W m^−2^ UVC irradiation; (vii) Post-AT, exposed to 0 W m^−2^ UVC irradiation and after that treated with 100 nM AT; (viii) UVC1+AT, exposed to 0.6 W m^−2^ UVC irradiation and after that treated with 100 nM AT; (ix) UVC2+AT, exposed to 1.2 W m^−2^ UVC irradiation and after that treated with 100 nM AT. Fifteen days after UVC exposure (both pre- and post-treated by AT), the plants were harvested for further morpho-physiological and biochemical analysis.

### 4.3. Growth Parameter Measurements

The dry weight of shoots was collected before UVC stress (W1) (at a time; T1) and at the harvest time (W2) (at a time; T2) and used for determination of RGR at different treatments based on the following equation: RGR = [W2-W1]/[T2-T1] [85]. Immediately after harvesting, the SFW and RFW of tomato plants were measured. After that, the plants were kept at 80 °C for 48 h, and the SDW and RDW were recorded. RWC, WLR, RT, and ELWR were analyzed using freshly harvested tomato leaves based on the method of Clarke et al. [86]. The epicuticular wax of fresh tomato leaves was determined following the method of Kakani et al. [46].

### 4.4. Photosynthetic Pigments Content Determination

For the extraction of photosynthetic pigments, the leaf samples were soaked in 5 mL of 95% ethyl alcohol overnight. The contents of photosynthetic pigments (Chl-*a*, Chl-*b*, and Car) were determined according to the method developed by Lichtenthaler and Wellburn [87].

### 4.5. SC, SP, FAA, and Pro Content Determination

The method based on anthrone-sulfuric acid, as described by Fales [88] and Schlegel [89], was employed to determine the contents of SC and starch, respectively. The method of Lowry et al. [90] was used to determine SP content by using bovine serum albumin as a standard. The FAA content was determined following the method of Moore and Stein [91]. The procedure of Bates et al. [92] was followed to estimate the Pro content.

### 4.6. Oxidative Stress Markers Determination

Several ROS, such as O_2_^•−^, H_2_O_2_, and ^•^OH, contents of tomato leaves were measured following the protocols of Mukherjee and Choudhuri [93], Elstner and Heupel [94], and Halliwell et al. [95], respectively. Lipid peroxidation was detected in leaves of tomato plants using the thiobarbituric acid reaction by monitoring MDA formation as explained by Rao and Sresty [96] with some modifications. LOX activity was measured according to Minguez-Mosquera et al. [97]. MG was estimated based on the method of Gilbert and Brandt [98].

### 4.7. Non-Enzymatic Antioxidants and Secondary Metabolites Estimation

For extraction of AsA and GSH, 0.5 g fresh leaves were ground with 5.0% trichloroacetic acid and centrifuged at 11,500 × *g* for 15 min at 4 °C, and the supernatant was utilized for quantification of AsA and GSH according to the protocols of Jagota and Dani [99] and Ellman [100], respectively. To determine the content of α-tocopherol, 0.2 g fresh leaves were grounded in 8.0 mL chloroform, centrifuged for 15 min at 4 °C; the supernatant was collected, and the measurement was performed following the protocol of Kivcak and Mert [101] using 2,2’-dipyridyl and ferric chloride reagents. The absorbance was recorded at 522 nm. The TPC was determined based on the method of Kofalvi and Nassuth [102]. Methanolic extract of fresh leaves was used for the detection of flavonoid according to Zou et al. [103], and the anthocyanins were detected based on the method of Krizek et al. [104].

### 4.8. Enzymatic Antioxidants Extraction and Assay

Fresh leaves of tomato plants were homogenized with potassium phosphate buffer (pH 7.8) containing ethylenediaminetetraacetic acid (EDTA) and polyvinylpyrrolidone, centrifuged at 11,500 × *g* for 30 min at 4 °C. The supernatant was collected and used for the determination of SOD (EC 1.15.1.1), CAT (EC 1.11.1.6), APX (EC 1.11.1.11), PPO (EC 1.10.3.1), and PAL (EC 4.3.1.5). The protein content of the supernatant was evaluated by the method of Lowry et al. [90].

SOD activity was quantified by following the autoxidation of epinephrine as mentioned by Misra and Fridovich (1972) in a medium containing sodium carbonate buffer (pH 10.2), EDTA, enzyme extract, and epinephrine. The change in absorbance was monitored at 480 nm. CAT activity was detected by monitoring the consumption of H_2_O_2_ [105], and the decrease in absorbance was recorded at 240 nm. APX activity was assessed by monitoring the oxidation of ascorbate at 290 nm in the presence of H_2_O_2_ [106]. PPO activity was determined following the protocol of Kumar and Khan [107] by monitoring the purpurogallin production at 495 nm. PAL activity was examined by the protocol of Havir and Hanson [108] with minor modification by incubation of 1000 µL of extract, 2000 µL of 80 mM borate buffer (pH 8.9), and 30 mM phenylalanine for 1 h at 30 °C, and then 1.5 mL of 2 M HCl was added where the content of trans-cinnamic acid was recorded at 290 nm. The activities of SPO and IPO were measured after the extraction of the enzymes from leaves according to the published method of Ghanati et al. [109].

### 4.9. H_2_S, Cysteine, Nitric Oxide Content, Nitrate Reductase Activity, AT, Allantoate, and Organic Acid Determination

The H_2_S and Cys contents of artichoke leaves were estimated based on the method of Nashef et al. [110] and Gaitonde [111], respectively. NO content was quantified through the method explained by Ding et al. [112] and Hu et al. [113]. The activity of NR was quantified in fresh leaves following the method of Downs et al. [114]. Foliar content of endogenous AT and allantoate was determined according to Voges and Van Der Drift [115]. The content of organic acid was measured by acid-base titration as recommended by the method of Zhang et al. [116].

### 4.10. Statistical Analysis

Data were subjected to a two-way analysis of variance (ANOVA) where the 1st factor is UVC stress (having three levels: control, UVC1, and UVC2) and the 2nd factor is AT treatments (having three levels: control, pre-AT, and post-AT) (see F-statistics and *P* values in Table S4), and followed by Tukey’s test (*p* < 0.05) using the “multcompView” package of the statistical programming language R 3.6.1. The numerical data in the table are presented as means ± standard deviation (SD) of five replicates for each treatment. The “*pheatmap()*” function of pheatmap package of R 3.6.1 was used for heatmap and hierarchical clustering considering Euclidean distances preparation with the normalized mean values. The cut-off of the heatmap and hierarchical clustering was performed by using the command “*cutree*” as 2. The packages ggplot2, factoextra, and FactoMineR of R 3.6.1 were used to perform principal component analysis (PCA) where the “*PCA()*” and “*fviz_pca_biplot()*” functions were used to perform PCA and visualize the PCA as biplot, respectively.

## 5. Conclusions

**Figure 7:**
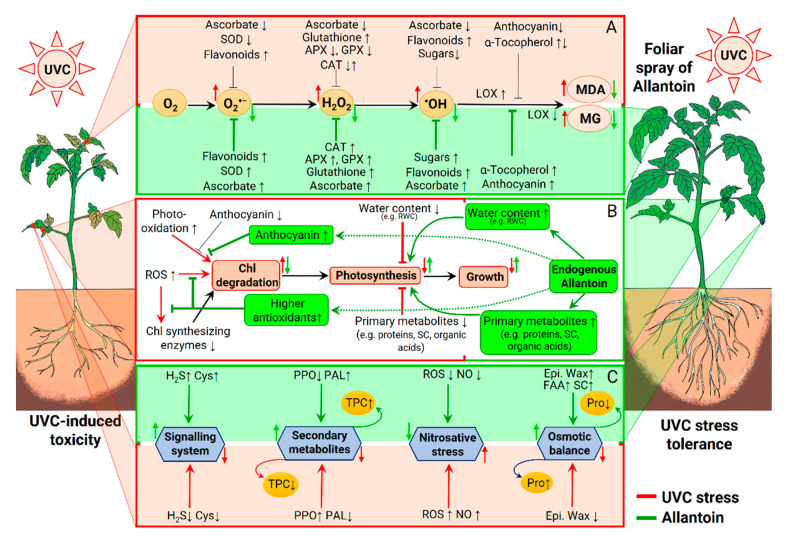
The probable mechanistic roles of allantoin (AT) in alleviating the adverse effects of UVC stress in tomato plants. Foliar application of AT positively altered several physio-biochemical processes in tomato plants. (**A**) UVC stress induces higher reactive oxygen species (ROS, O_2_^•−^, H_2_O_2_, and ^•^OH) accumulation by lowering several non-enzymatic and enzymatic antioxidants, and ROS induces higher lipoxygenase (LOX) activity and causes lipid peroxidation; as a result, tomato plants accumulate higher amounts of malondialdehyde (MDA) and methylglyoxal (MG). However, exogenous AT treatment enhances non-enzymatic and enzymatic antioxidants and lowers LOX activity and ultimately reduced MDA and MG accumulation. (**B**) Exposure to UVC stress causes chlorophyll (Chl) degradation by enhancing ROS that deactivates Chl synthesizing enzymes or directly oxidize Chl pigments. UVC stress also lowers the anthocyanin content and accelerates photo-oxidation-induced Chl degradation. The efficiency of photosynthesis is reduced in the UVC-stressed tomato plants due to the Chl degradation and decreased water content and primary metabolites in plant leaves, leading to growth retardation. On the other hand, exogenous AT enhances the endogenous allantoin content and upregulates anthocyanin content, primary metabolites, and water content. Moreover, AT-mediated higher antioxidants inhibits ROS accumulation and protects Chl pigments. Thus, exogenous AT enhances the growth of tomato plants under UVC stress. (**C**) UVC stress lowers H_2_S and Cys contents and impairs plant signaling networks. The decreased phenylalanine ammonia-lyase (PAL) and increased polyphenol oxidase (PPO) activity induced by UVC cause less secondary metabolite contents. Enhanced ROS and NO simultaneously cause nitrosative stress, and reduced epicuticular wax causes osmotic stress in UVC-stressed tomato plants. Application of exogenous AT improves the signaling system, secondary metabolism, osmotic balance, and mitigates nitrosative stress by altering the aforementioned processes. SC, soluble carbohydrate content; FAA, free amino acid content; TPC, total phenolic compound content.

The results of this experiment led us to conclude that excessive UVC is a phytotoxic agent, and AT could be a useful chemical for reducing extreme UVC-induced adverse effects in tomato by modulating several physio-biochemical processes, as summarized in Figure 7. Particularly, AT-induced UVC stress tolerance could be attributed to (i) improvement of plant water retention via regulation of osmolytes, (ii) protection of photosynthetic pigments, (iii) efficient activity of enzymatic antioxidants to reduce ROS-induced membrane destruction, and (iv) accumulation of non-enzymatic antioxidants and secondary metabolites and its biosynthetic enzymes. Our findings suggest that the potential role of AT is effective in UV stress mitigation in tomato hybrid cultivar “F1-841” if we pretreat the plants. Further investigation, at the genetic and molecular level and field condition, is urgent to reveal the potentiality of AT-induced UVC stress mitigation in other varieties of tomato plants and perhaps in other crops.

## Figures and Tables

**Table 1:** Effects of pre-treatment and post-treatment of allantoin (AT) on growth and photosynthetic pigments in tomato plants grown 15 days in the presence or absence of UVC stress.

Treatments	RGR	SFW (g)	RFW (g)	SDW (g)	RDW (g)	Chl-*a* Content	Chl-*b* Content	Chl-*a+b* Content	Car Content
C	0.050 ± 0.002 ^d^	8.64 ± 0.32 ^f^	3.08 ± 0.11 ^d^	0.933 ± 0.03 ^g^	0.206 ± 0.007 ^e^	2.43 ± 0.09 ^f^	0.670 ± 0.02 ^d^	3.11 ± 0.11 ^f^	1.30 ± 0.04 ^e^
UVC1	0.025 ±0.003 ^a,b^	4.59 ± 0.17 ^a^	2.10 ± 0.07 ^a^	0.490 ± 0.01 ^a,b^	0.155 ± 0.005 ^a^	1.37 ± 0.05 ^a,b^	0.559 ± 0.02 ^a^	1.93 ± 0.07 ^a,b^	0.745 ± 0.02 ^a^
UVC2	0.017 ± 0.000 ^a^	3.49 ± 0.12 ^b^	1.03 ± 0.03 ^b^	0.361 ± 0.01 ^c^	0.075 ± 0.002 ^b^	0.893 ± 0.03 ^c^	0.344 ± 0.01 ^b^	1.23 ± 0.04 ^c^	0.494 ± 0.01 ^b^
Pre-AT	0.076 ± 0.004 ^c^	11.77 ± 0.11 ^c^	4.56 ± 0.04 ^c^	1.927 ± 0.01 ^d^	0.230 ± 0.002 ^c^	3.18 ± 0.03 ^d^	0.874 ± 0.008 ^c^	4.06 ± 0.04 ^d^	1.55 ± 0.01 ^c^
AT+ UVC1	0.044 ± 0.003 ^d^	7.40 ± 0.04 ^d^	2.83 ±0.01 ^d^	0.726 ± 0.00 ^e^	0.224 ± 0.001 ^c^	2.38 ± 0.01 ^e,f^	0.662 ± 0.004 ^d^	3.04 ± 0.01 ^e,f^	2.34 ± 0.01 ^d^
AT+ UVC2	0.029 ± 0.001 ^b^	6.40 ± 0.33 ^e^	1.84 ± 0.09 ^a^	0.608 ± 0.03 ^a,e^	0.113 ± 0.005 ^d^	2.06 ± 0.10 ^e^	0.713 ± 0.03 ^d^	2.77 ± 0.14 ^e^	1.37 ± 0.07 ^e^
Post-AT	0.078 ± 0.003 ^c^	11.11 ± 0.57 ^c^	3.95 ± 0.20 ^e^	2.088 ± 0.10 ^f^	0.221 ± 0.011 ^c,e^	3.35 ± 0.17 ^d^	0.878 ± 0.04 ^c^	4.23 ± 0.21 ^d^	1.25 ± 0.06 ^e^
UVC1+AT	0.031 ± 0.002 ^b^	5.63 ± 0.21 ^e^	2.43 ± 0.09 ^f^	0.589 ± 0.02 ^a,b^	0.168 ± 0.006 ^a^	1.66 ± 0.06 ^a^	0.548 ± 0.003 ^a^	2.20 ± 0.06 ^a^	1.31 ± 0.05 ^e^
UVC1+AT	0.021 ± 0.002 ^a^	4.31 ± 0.15 ^a^	1.48 ± 0.05 ^g^	0.471 ± 0.01 ^b,c^	0.093 ± 0.003 ^f^	1.28 ± 0.04 ^b^	0.443 ± 0.004 ^e^	1.72 ± 0.04 ^b^	0.539 ± 0.01 ^b^

Values are means ± standard deviation (SDs) (*n* = 5). Means followed by the same letter are non-significant among the treatments within the same column at *p* ≤ 0.05 considering Tukey’s test. RGR, relative growth rate, SFW, shoot fresh weight; RFW, root fresh weight; SDW, shoot dry weight; RDW, root dry weight; chlorophyll (Chl)-*a* (mg g^−1^ FW); Chl-*b* (mg g^−1^ FW); Chl-*a+b* (mg g^−1^ FW), and carotenoids (Car, mg g^−1^ FW). “C”, no AT and grown under non-stress condition; “UVC1”, exposed to 0.6 W m^−2^ UVC irradiation; “UVC2”, exposed to 1.2 W m^−2^ UVC irradiation; “Pre-AT”, pretreated with 100 nM AT and after that exposed to 0 W m^−2^ UVC irradiation; “AT+UVC1”, pretreated with 100 nM AT and after that exposed to 0.6 W m^−2^ UVC irradiation; “AT+UVC2”, pretreated with 100 nM AT and after that exposed to 1.2 W m^−2^ UVC irradiation; “Post-AT”, exposed to 0 W m^−2^ UVC irradiation and after that post-treated with 100 nM AT; “UVC1+AT”, exposed to 0.6 W m^−2^ UVC irradiation and after that post-treated with 100 nM AT; “UVC2+AT”, exposed to 1.2 W m^−2^ UVC irradiation and after that post-treated with 100 nM AT.

## Data Availability

No new data were created or analyzed in this study. Data sharing is not applicable to this article.

## Supplementary Materials

The following are available online at https://www.mdpi.com/2223-7747/10/1/11/s1, Supporting Materials and Methods, Figure S1: (A) Effects of different levels of UVC on phenotypes of tomato plants. (B) Phenotypic appearance of different concentrations of allantoin pre-treated tomato plants exposed to UVC stress; Figure S2: Leaf phenotypic appearance of allantoin (100 nM) pre-treated tomato plants in absence or presence of UVC (0.6 W m^−2^) stress. Table S1: Effect of different doses of UVC stress on tomato plants; Table S2: Effect of different doses of allantoin (AT) in tomato plants under UVC stress; Table S3: Effects of pre-treatment and post-treatment of allantoin (AT) on water relation parameters in tomato plants grown 15 days in the presence or absence of UVC stress; Table S4: F-statistics and *p* values of all studied parameters from two-way ANOVA.
