# Supplementary material for: Mechanistic Insight of Allantoin in Protecting Tomato Plants Against Ultraviolet C Stress
Source: Plants (Basel). 2020 Dec 23;10(1):11. doi: 10.3390/plants10010011 (PMC7824269; doi:10.3390/plants10010011)
Supplement: Supplementary file 1 [file plants-10-00011-s001.pdf]

Supplementary file

Supporting Materials and Methods

Determination of the lethal dose of UVC and optimal dose of AT

Plastic pots lined with a plastic bag containing 1 kg clay soil were used for sub-culturing of 40 day-old tomato transplants (3 plants pot^{-1}). The pots were divided into five lots (five pots lot^{-1}) receiving different irradiance of 0, 0.3, 0.6, 1.2, 1.8 W m^{-2}. The control group (not treated with UVC) was grown under natural conditions. Plant height (PH) and root length (RL) were measured after 7 days after treatment. Shoot and root dry matter content was measured by drying the plant material at 80 °C for 48 h. AT and allantoate contents were determined in leaves samples.

Different concentrations of AT (10^{-2}, 10^{-4}, 10^{-6}, 10^{-7}, $10^{-8,}$ and 10^{-10} M) were applied to 40 day-old tomato plants as a foliar spray. Each plant was sprayed with 20 mL of AT solution at the desired concentration. Then the plants were subjected to UVC dose at the first irradiance does (provided from the previous experiment) caused a reduction of foliar AT and allantoate of tomato plants. The UVC lamps were stabilized by turning on lamps at 11:00 AM for the calculated time of each irradiance treatment and then closed, this process was conducted for three days. One week after the last UVC dose, the plants were harvested for morphological parameters as well as foliar AT and allantoate contents.

Supplementary Figures and Tables

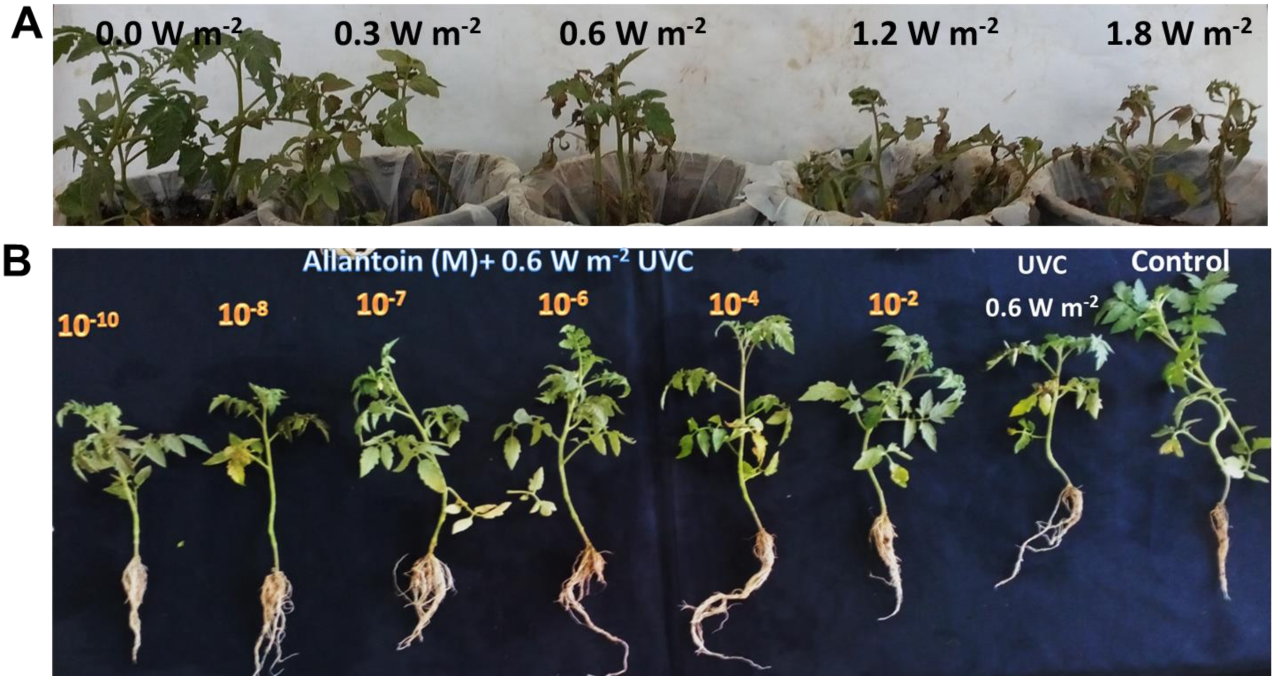

Figure S1. (**A**) Effects of different levels of UVC on phenotypes of tomato plants. (**B**) Phenotypic appearance of different concentrations of allantoin pre-treated tomato plants exposed to UVC stress.

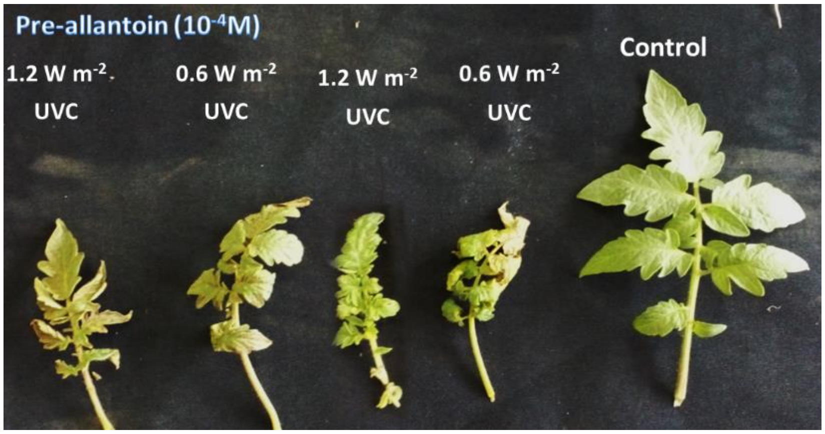

Figure S2. Leaf phenotypic appearance of allantoin (100 nM) pre-treated tomato plants in absence or presence of UVC (0.6 W m^{-2}) stress.

Table S1. Effect of different doses of UVC stress on tomato plants

UVC exposure	SFW (g)	RFW (g)	SDW (g)	RDW (g)	Allantoin (mg g^{-1} FW)	Allantoate (mg g^{-1} FW)
0 W m^{-2}	4.973^{a}	1.633^{a}	0.965^{a}	0.237^{a}	524.224^{a}	634.894^{a}
0.3 W m^{-2}	3.286^{b}	1.180^{b}	0.508^{b}	0.195^{b}	560.434^{a}	646.980^{a}
0.6 W m^{-2}	2.156^{bc}	0.863^{c}	0.439^{b}	0.080^{c}	447.467^{b}	517.441^{b}
1.2 W m^{-2}	2.053^{bc}	0.627^{d}	0.363^{c}	0.070^{c}	370.215^{c}	448.437^{c}
1.8 W m^{-2}	1.318^{c}	0.330^{e}	0.242^{d}	0.038^{d}	286.986^{d}	330.974^{d}

Values are means of five replicates ($n = 5$). Means followed by the same letter are non-significant among the treatments within the same column at $P \leq 0.05$ considering Tukey's test. SFW, shoot fresh weight; RFW, root fresh weight; SDW, shoot dry weight; RDW, root dry weight;

Table S2. Effect of different doses of allantoin (AT) in tomato plants under UVC stress

Treatments	SFW (g)	RFW (g)	Allantoin (mg g^{-1} FW)	Allantoate (mg g^{-1} FW)
Control	9.247^{a}	2.610^{cd}	629.558^{d}	751.088^{c}
0.6 W m^{-2} (UV0.6)	5.071^{d}	1.972^{d}	504.000^{e}	679.350^{d}
UV0.6+10^{-2} M AT	6.423^{bcd}	4.941^{a}	673.229^{cd}	793.626^{bc}
UV0.6+10^{-4} M AT	**7.340^{b}**	3.381^{b}	**846.598^{a}**	**945.221^{a}**
UV0.6+10^{-6} M AT	6.754^{bc}	2.513^{cd}	736.439^{b}	829.942^{b}
UV0.6+10^{-7} M AT	5.768^{cd}	2.884^{bc}	717.218^{bc}	783.217^{bc}
UV0.6+10^{-8} M AT	5.427^{cd}	2.116^{d}	691.323^{bc}	766.627^{bc}
UV0.6+10^{-10} M AT	5.024^{d}	2.011^{d}	686.118^{bcd}	759.509^{bc}

Values are means of five replicates ($n = 5$). Means followed by the same letter are non-significant among the treatments within the same column at $P \leq 0.05$ considering Tukey's test. SFW, shoot fresh weight; RFW.

Table S3. Effects of pre-treatment and post-treatment of allantoin (AT) on water relation parameters in tomato plants grown 15 days in the presence or absence of UVC stress.

Treatments	WLR	ELWR (%)	RT (g H_2O min^{-1} cm^{2} × 10^{5})
**C**	**4.80±0.17^{e}**	**83.95±0.84^{cd}**	15.10±1.99^{efg}
**UVC1**	3.64±0.13^{ab}	71.72±1.39^{a}	19.42±0.66^{ab}
**UVC2**	2.98±0.11^{c}	57.56±0.73^{b}	24.91±1.52^{c}
**Pre-AT**	5.63±0.05^{d}	88.76±0.68^{c}	10.76±0.30^{d}
**AT+ UVC1**	4.42±0.17^{ef}	82.32±0.58^{d}	14.26±0.87^{ef}
**AT+ UVC2**	3.73±0.29^{ab}	79.52±0.59^{d}	17.00±1.80^{aeg}
**Post-AT**	4.76±0.48^{e}	87.95±1.67^{c}	12.98±0.32^{df}
**UVC1+AT**	4.05±0.15^{af}	79.52±0.59^{d}	17.94±0.66^{abg}
**UVC1+AT**	3.13±0.11^{bc}	61.53±4.05^{b}	20.72±0.92^{b}

Values are means ± standard deviation (SDs) (n = 5). Means followed by the same letter are non-significant among the treatments within the same column at $P \leq 0.05$ considering Tukey's test. WLR, water loss rate; ELWR, excised leaf water retention; RT, residual transpiration. 'C', no AT and grown under non-stress condition; 'UVC1', exposed to 0.6 W m^{-2} UVC irradiation; 'UVC2', exposed to 1.2 W m^{-2} UVC irradiation; 'Pre-AT', pretreated with 100 nM AT and after that exposed to 0 W m^{-2} UVC irradiation; 'AT+UVC1', pretreated with 100 nM AT and after that exposed to 0.6 W m^{-2} UVC irradiation; 'AT+UVC2', pretreated with 100 nM AT and after that exposed to 1.2 W m^{-2} UVC irradiation; 'Post-AT', exposed to 0 W m^{-2} UVC irradiation and after that post-treated with 100 nM AT; 'UVC1+AT', exposed to 0.6 W m^{-2} UVC irradiation and after that post-treated with 100 nM AT; 'UVC2+AT', exposed to 1.2 W m^{-2} UVC irradiation and after that post-treated with 100 nM AT.

Table S4. F-statistics and *P* values of all studied parameters from two-way ANOVA

Parameters	F-value			*P* value		
	Factor 1: UVC	Factor 2: AT	Interaction: UVC×AT	Factor 1: UVC	Factor 2: AT	Interaction: UVC×AT
RGR	661.66	110.30	18.00	<0.0001	<0.0001	<0.0001
SFW	1116.29	259.20	9.30	<0.0001	<0.0001	<0.0001
RFW	1384.93	238.12	14.01	<0.0001	<0.0001	<0.0001
SDW	2143.23	388.89	168.84	<0.0001	<0.0001	<0.0001
RDW	1027.45	120.46	15.39	<0.0001	<0.0001	<0.0001
Chl *a*	900.01	315.99	32.78	<0.0001	<0.0001	<0.0001
Chl *b*	424.62	216.98	46.13	<0.0001	<0.0001	<0.0001
Carotenoids	647.80	1148.21	200.02	<0.0001	<0.0001	<0.0001
SP	65.63	207.57	3.25	<0.0001	<0.0001	0.036
SC	694.87	270.09	20.54	<0.0001	<0.0001	<0.0001
FAA	157.32	30.71	4.31	<0.0001	<0.0001	0.013
Proline	1214.53	1290.95	495.11	<0.0001	<0.0001	<0.0001
WLR	143.82	30.43	1.16	<0.0001	<0.0001	0.362
ELWR	332.63	120.95	29.11	<0.0001	<0.0001	<0.0001
RT	103.89	55.75	2.29	<0.0001	<0.0001	0.099
RWC	27.87	26.60	5.08	<0.0001	<0.0001	0.006
Wax	185.48	180.48	1.85	<0.0001	<0.0001	0.164
AsA	24.16	222.05	16.04	<0.0001	<0.0001	<0.0001
GSH	64.65	40.65	8.90	<0.0001	<0.0001	<0.0001

α-tocopherol	0.80	248.53	13.95	0.464	<0.0001	<0.0001
Anthocyanin	5 16.27	162.08	17.09	<0.0001	<0.0001	<0.0001
Flavonoids	172.31	230.26	68.53	<0.0001	<0.0001	<0.0001
TPC	844.89	580.97	50.47	<0.0001	<0.0001	<0.0001
MDA	303.64	71.54	34.78	<0.0001	<0.0001	<0.0001
LOX	2174.02	418.35	252.91	<0.0001	<0.0001	<0.0001
MG	3029.33	302.67	96.81	<0.0001	<0.0001	<0.0001
Allantoate	106.68	675.16	150.11	<0.0001	<0.0001	<0.0001
Allantoin	237.76	739.64	138.81	<0.0001	<0.0001	<0.0001
OA	52.22	9.94	4.25	<0.0001	<0.0001	0.014
H_2S	98.96	505.95	75.73	<0.0001	<0.0001	<0.0001
NO	2009.02	234.15	129.33	<0.0001	<0.0001	<0.0001
$O_2^{\bullet -}$	534.04	205.58	26.17	<0.0001	<0.0001	<0.0001
$^{\bullet}OH$	630.90	56.95	16.44	<0.0001	<0.0001	<0.0001
H_2O_2	629.37	613.76	288.05	<0.0001	<0.0001	<0.0001
CAT	334.83	634.75	64.79	<0.0001	<0.0001	<0.0001
APX	10.97	1543.27	35.66	0.001	<0.0001	<0.0001
IPO	1190.29	329.10	132.84	<0.0001	<0.0001	<0.0001
SPO	898.43	265.07	95.92	<0.0001	<0.0001	<0.0001
PPO	1881.39	170.87	51.66	<0.0001	<0.0001	<0.0001
GPX	749.92	1148.56	290.86	<0.0001	<0.0001	<0.0001
GST	606.92	328.81	89.22	<0.0001	<0.0001	<0.0001
SOD	216.07	1198.34	52.90	<0.0001	<0.0001	<0.0001
PAL	1040.96	338.98	81.21	<0.0001	<0.0001	<0.0001
NR	55.56	23.65	1.22	<0.0001	<0.0001	0.337
